# Eradication of *Helicobacter pylori* and Gastric Cancer: A Controversial Relationship

**DOI:** 10.3389/fmicb.2021.630852

**Published:** 2021-02-04

**Authors:** Mariagrazia Piscione, Mariangela Mazzone, Maria Carmela Di Marcantonio, Raffaella Muraro, Gabriella Mincione

**Affiliations:** Department of Innovative Technologies in Medicine and Dentistry, University “G. d’Annunzio” of Chieti–Pescara, Chieti, Italy

**Keywords:** gastric cancer, *Helicobacter pylori*, inflammation, gut microbiota, eradication

## Abstract

Worldwide, gastric cancer (GC) represents the fifth cancer for incidence, and the third as cause of death in developed countries. Indeed, it resulted in more than 780,000 deaths in 2018. *Helicobacter pylori* appears to be responsible for the majority of these cancers. On the basis of recent studies, and either alone or combined with additional etiological factors, *H. pylori* is considered a “type I carcinogen.” Over recent decades, new insights have been obtained into the strategies that have been adopted by *H. pylori* to survive the acidic conditions of the gastric environment, and to result in persistent infection, and dysregulation of host functions. The multistep processes involved in the development of GC are initiated by transition of the mucosa into chronic non-atrophic gastritis, which is primarily triggered by infection with *H. pylori*. This gastritis then progresses into atrophic gastritis and intestinal metaplasia, and then to dysplasia, and following Correa’s cascade, to adenocarcinoma. The use of antibiotics for eradication of *H. pylori* can reduce the incidence of precancerous lesions only in the early stages of gastric carcinogenesis. Here, we first survey the etiology and risk factors of GC, and then we analyze the mechanisms underlying tumorigenesis induced by *H. pylori*, focusing attention on virulence factor CagA, inflammation, oxidative stress, and ErbB2 receptor tyrosine kinase. Moreover, we investigate the relationships between *H. pylori* eradication therapy and other diseases, considering not only cardia (upper stomach) cancers and Barrett’s esophagus, but also asthma and allergies, through discussion of the “hygiene hypothesis. ” This hypothesis suggests that improved hygiene and antibiotic use in early life reduces microbial exposure, such that the immune response does not become primed, and individuals are not protected against atopic disorders, asthma, and autoimmune diseases. Finally, we overview recent advances to uncover the complex interplay between *H. pylori* and the gut microbiota during gastric carcinogenesis, as characterized by reduced bacterial diversity and increased microbial dysbiosis. Indeed, it is of particular importance to identify the bacterial taxa of the stomach that might predict the outcome of gastric disease through the stages of Correa’s cascade, to improve prevention and therapy of gastric carcinoma.

## Introduction

Gastric cancer (GC) is a commonly diagnosed cancer, as number five worldwide, and it represents the third most-common cause of cancer-related deaths in developed countries. In this review, we aim to analyze the diffusion of GC and its main risk factors, with a focus on the role of *Helicobacter pylori* infection. Indeed, *H. pylori* is believed to be closely involved in the chronic inflammation behind duodenal ulcers and gastric diseases, and therefore it is crucial to understand how *H. pylori* causes the progression from acute inflammation of the mucosa to GC.

### Gastric Cancer Incidence and Classification

According to GLOBOCAN 2018 data, the worldwide cumulative risk of developing GC from birth to 74 years old is 1.87% in males and 0.79% in females. However, the overall incidence rates for GC have decreased markedly over the past 75 years, although mortality remains high in Japan, China, Chile, and Ireland (Rawla and Barsouk, 2019). Migrants from countries of high incidence to low incidence have been shown to maintain susceptibility to GC, while their children follow a risk that is more similar to that of the new homeland. Environmental exposure and dietary carcinogens are considered to be the most likely risk factors (Rawla and Barsouk, 2019).

It is well known that mortality rates from GC are high in males living in central Asia and Latin America. As this incidence is accompanied by survival rates that are low, and as there are few treatment options, particularly in developing countries, decreased incidence of GC would appear to be the key to reduced mortality. Over the past 50 years, changes have been seen for food preservation, with less smoking and processing of meat, and greater availability of fresh fruit and vegetables. Indeed, incidence of non-cardia GCs, in particular, has decreased more recently because of the decline in *H. pylori* infection rates (Rawla and Barsouk, 2019).

**FIGURE 1:**
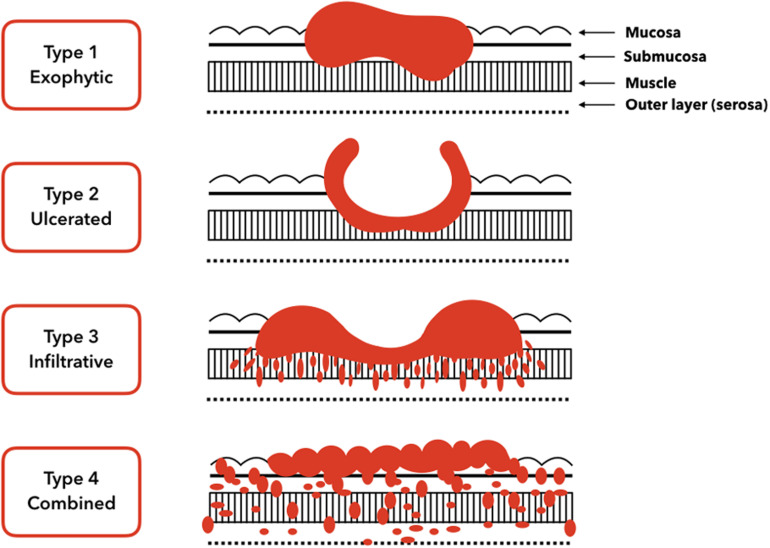
Gross tumor morphology of gastric carcinomas. Type 1 is a classical superficial tumor whereas from type 2 to type 4 tumors are advanced.

Gastric carcinomas can be classified clinically as early or advanced; moreover, their histology can be divided into subtypes based on the morphological components (Mayer, 2017). Early gastric carcinomas are defined as invasive carcinomas of the mucosa or submucosa (irrespective of tumor size) that can be accompanied by lymph node metastases. Early gastric carcinomas are generally small, at 2–5 cm, and usually located in surfaces with little curvature, such as around the angularis. However, some early gastric carcinomas can be multifocal. In contrast, advanced gastric carcinomas have been defined as exophytic, ulcerated, infiltrative, and combined (Figure 1).

The prognosis for early gastric carcinomas is relatively good, with 5 years survival rates of around 90%. In contrast, advanced gastric carcinomas that invade the muscularis propria or deeper have much worse prognosis, with 5 years survival rates of ≤60% (Japanese Gastric Cancer Association, 2011).

Gastric cancers can also be histologically classified as carcinomas, adenocarcinomas, gastrointestinal stromal tumors, lymphomas, and leiomyosarcomas. The majority of these are gastric adenocarcinomas, and they can be further subdivided into diffuse and intestinal types. There is little cell cohesion in diffuse gastric adenocarcinomas, such that the individual cells do not form a solid mass, but instead show “disorderly” infiltration of the stomach wall. Conversely, the intestinal type shows a cohesive pattern in which the neoplastic cells form gland-like tubular structures (Mayer, 2017).

Young patients generally develop diffuse carcinomas, throughout the stomach, and including the cardia (upper area of the stomach, close to the esophageal opening). This results in what is known as *linitis plastica*, where the stomach wall loses its distensibility, with a “leather bottle” appearance. The intestinal lesion types are frequently ulcerative; they are usually in the antrum, in areas of the stomach that show less curvature, and they often develop after long precancerous processes (Mayer, 2017). *H. pylori* infection is strongly associated with both of these types of gastric adenocarcinomas. However, incidence of the intestinal type of gastric adenocarcinoma has declined over the last 60 years, which has not been seen for the diffuse type (Houghton and Wang, 2005). Overall, this decline in the intestinal type appears to represent the decreased incidence of GC for most developed countries, which has been attributed to their greatly reduced prevalence of *H. pylori* (Houghton and Wang, 2005).

### Gastric Cancer Etiology

On the basis of its status as a particularly important risk factor in the development of GC, *H. pylori* has been classified as a type I carcinogen by the International Agency for Research on Cancer and by the World Health Organization (IARC Working Group on the Evaluation of Carcinogenic Risks to Humans, 2012). The mechanism that leads to GC is not completely understood yet; however, the effects of *H. pylori* on GC appear to be multifactorial (Valenzuela et al., 2015). *H. pylori* can cause chronic gastritis, along with loss of gastric acidity, which leads to bacterial growth in the stomach (Rawla and Barsouk, 2019). Nowadays, the effects that eradication of *H. pylori* might have on the risk for GC and esophageal cancer are debatable.

**FIGURE 2:**
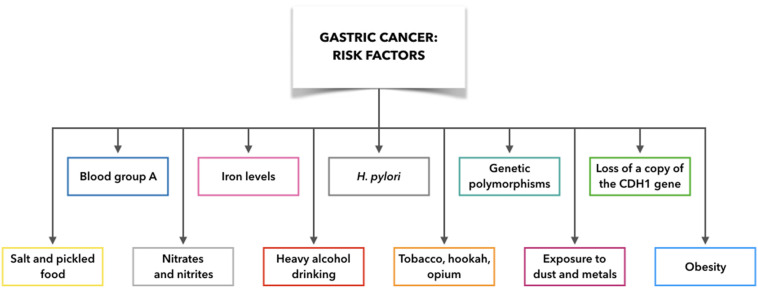
Gastric cancer risk factors: what make people more likely to get gastric cancer. CDH1: cadherin 1.

Indeed, GC is not only associated with *H. pylori* infection, but also with several additional etiological factors and their combinations with *H. pylori* infection, such as gastric ulcers and adenomatous polyps, blood group, gene mutations, obesity, exposure to dust and metals, tobacco, hookah, and opium use, and alcohol consumption (Figure 2). For example, blood group A is associated with high incidence of GC compared to blood group O, which appears to arise from variations in mucous secretion (Rawla and Barsouk, 2019). Moreover, if one copy of the cadherin 1 (*CDH1*) gene is lost, this can result in development of diffuse GC, which is also connected with prostate cancer, colorectal cancer, and lobular breast cancer. Asian populations also show interleukin (IL)-10 and IL-17 polymorphisms that are associated with elevated risk of neoplasia (Rawla and Barsouk, 2019). In this regard, Zhuang et al. (2010) found that the IL-10 -592 promoter polymorphism may be associated in Asians with gastric cancer development (Zhuang et al., 2010). Moreover, Wu et al. demonstrated that the polymorphism of IL-17F 7488 is involved in susceptibility to gastric cancer associated with IL-17-induced inflammation in response to *H. pylori* infection (Wu et al., 2010). These results indicate that genetic variations in inflammation-related genes may play a role in the outcome of *H. pylori* infection and development of gastric lesions.

Although some risk factors for GC are now well defined (e.g., tobacco, hookah, opium), it remains demanding to demonstrate any link between moderate alcohol consumption and GC. However, it is clear that heavy alcohol drinking can promote erosion of the gastric mucosa, with the consequent gastritis, which is the precursor to GC. Exposure to high temperature particulates, dust, and metals (e.g., chromium VI) is also implicated in non-cardia GC (Rawla and Barsouk, 2019).

A balanced diet is considered to be of importance to avoid gastritis and GC. Salt can erode the protective stomach mucosa, and cultures where diets are rich in salt and pickled foods (e.g., Japan) are associated with high rates of GC (Rawla and Barsouk, 2019). Furthermore, it has been demonstrated that if the sodium chloride concentrations change in the bacterial culture medium, *H. pylori* gene expression is remarkably altered (Loh et al., 2007, 2012). Moreover, Ganez et al. (2008) argued that *H. pylori* changed from a typical spiral shape to a more elongated one arranging chains (Ganez et al., 2008). In conclusion, it may be stated that high salt concentration produces a change in cell morphology and probably in pathogenetic mechanism.

Moreover, iron deficiency determines an increased risk for GC and gastrointestinal tract tumors (Pra et al., 2009). *H. pylori* colonization is correlated with hemorrhagic gastritis which causes iron deficiency (Yip et al., 1997). *H. pylori* colonization of gastric antrum leads to hypochlorhydria, resulting in reduced levels of ascorbic acid and subsequently a lower absorption of iron (Waring et al., 1996).

Furthermore, preserved meats are rich in N-nitroso compounds, while red meat from grain-fed animals shows particularly high saturated fats, with low levels of the protective omega-3 fatty acids. This can contribute to inflammatory processes, which can also increase the risk of GC. Higher intake of fruit and vegetables in case-control studies has been shown to be associated with reduced risk of GC, by over a third (Rawla and Barsouk, 2019). Obesity also strongly predisposes males and non-Asians, in particular, to GC. This condition can promote inflammation of the stomach mucosa through secretion of IL-6, tumor necrosis factor-α, and monocyte chemoattractant protein-1 (Rawla and Barsouk, 2019).

## Review Strategies and Literature Included

For this review, the PubMed database was used for the articles search. The keywords were: “*H. pylori* and gastric cancer” AND “*H. pylori* and treatment” AND “*H. pylori* and treatment and microbiota.” Papers in the English language that were published (or accepted for publication) between 2010 and 2020 were included, except for Correa’s “Human gastric carcinogenesis: a multistep and multifactorial process,” dated 1992. This publication remains a landmark in the literature on the theme of gastric carcinogenesis and *H. pylori*, and it has also been mentioned in numerous other studies. Further, all of the literature references were reviewed from each publication included; this provided additional articles that were not included in the initial screening.

**FIGURE 3:**
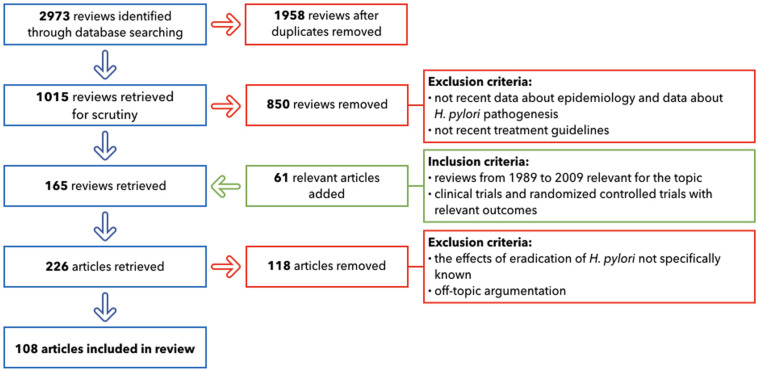
Flow chart of literature selection.

The primary search, after duplicates removed, provided 1,015 reviews. The following important step involved the selection of only the publications that included: up-to-date details on GC epidemiology; the most recent studies about *H. pylori* pathogenesis; the treatment guidelines widely used in 2019; and the relationship with other different diseases that can emerge after antibiotic therapies. This led to the inclusion of 165 reviews. 61 relevant articles were added: they included reviews from 1989 to 2009, crucial for the topic and clinical trials and randomized controlled trials with relevant outcomes. The following step for the selection excluded all of the papers where the effects of the eradication of *H. pylori* was not specifically known (118 removed). After applying these criteria, 108 papers provided the core literature for the current review (Figure 3).

## *Helicobacter Pylori*: An Introduction

The Nobel Prize for Medicine in 2005 was won by Marshall and Warren following their demonstration that *H. pylori* is involved in peptic ulcer disease (Wroblewski et al., 2010). This Gram-negative pathogen can colonize the gastric epithelium. *H. pylori* is spiral shaped, with from 3 to 5 polar flagellae that provide motility, and is urease, catalase, and oxidase positive. It lives in the mucus of the stomach, with the greater proportion adherent to the mucosa, while only a few bacterium cells will enter the mucosal cells, and their distribution is never systemic (Wroblewski et al., 2010). In addition, *H. pylori* expresses virulence factors that can affect host cell signaling pathways. A particularly important characteristic of *H. pylori* is that it can persist for decades in the gastric environment, as the host cannot eliminate the infection. Therefore, this pathogen can colonize the highly acidic environment of the stomach, where urea can be metabolized by its urease to ammonia, with the consequent generation of a neutral environment that surrounds the bacteria (Wroblewski et al., 2010).

*Helicobacter pylori* infections are found all over the world, with high rates of colonization typical of developed countries (Hooi et al., 2017). Humans are the only reservoir for *H. pylori*. Childhood acquisition is believed to occur for most infections, through fecal–oral and oral–oral transmission. Prevalence varies according to age, and in the United States some 50% of 60-years-olds are colonized, while 10% of children have contracted the infection. Children acquire the infection from their parents or from other children (Wroblewski et al., 2010).

Infection by *H. pylori* promotes a response in the tissue of the stomach, with superficial gastritis that involves mucosal infiltration by neutrophils and mononuclear cells. Although *H. pylori* has probably undergone adaptation to minimize stimulation of the host immune system, following its colonization there is a persistent immune response, with antibody production and further cell-mediated responses. As these responses do not appear to clear the *H. pylori*, and the immune system is then down-regulated, this results in *H. pylori* persistence (Ansari and Yamaoka, 2019). Further investigations are required to understand this activation of the immune system further, along with its relationship with the persistence of *H. pylori*.

The gastric inflammation is known to be associated with the risk of disease here, whereby antral gastritis has been linked to duodenal ulceration, and also pangastritis can lead to gastric ulceration and adenocarcinoma. The processes toward duodenal ulceration as a result of gastric colonization by *H. pylori* arise initially through *H. pylori-*induced inflammation. This then results in decreased numbers of the D cells that produce somatostatin, which inhibits gastrin release. This is seen as higher gastrin levels in patients who are *H. pylori* positive, and who thus have increased acid secretion (induced by gastrin) in the gastric corpus. The next stage for increased risk of duodenal ulcer remains under debate, with this increased acid secretion potentially contributing to formation of gastric metaplasia in the duodenum; this can also be colonized by *H. pylori*, to lead to further inflammation and ulceration (Ansari and Yamaoka, 2019). Gastric ulceration and gastric adenocarcinoma have a less well understood pathogenesis today, although it is known that these conditions can arise through pangastritis and corpus-predominant gastritis. Gastric ulcers are usually seen to occur where the antral and corpus-type mucosa join (Ansari and Yamaoka, 2019).

**FIGURE 4:**
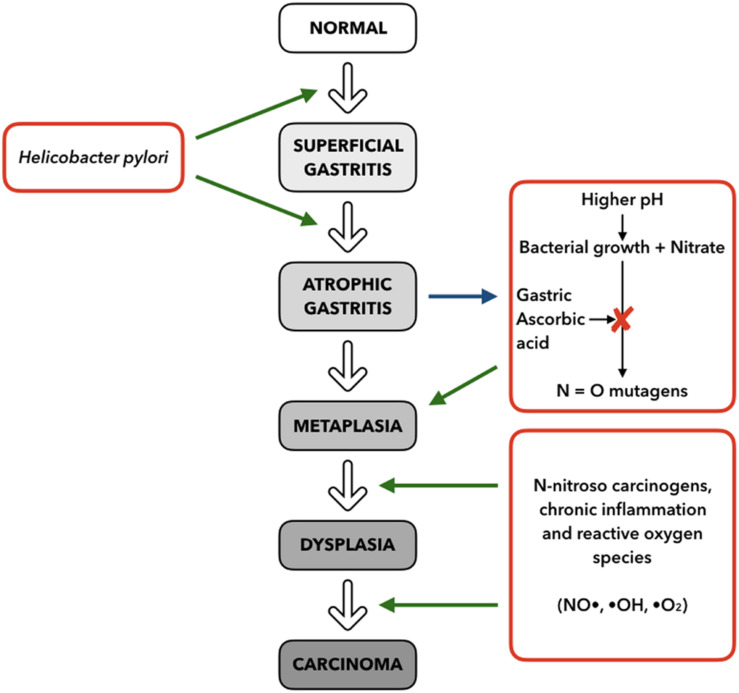
Multistep model for progression of gastric cancer, based on stimuli and mechanisms **(right and left)** interacting with the histopathologic Correa Cascade **(center)**, from superficial gastritis to carcinoma (adapted from Fox and Wang, 2001).

The development of GC is known to be a multistep process where the normal mucosa is transformed into non-atrophic gastritis. Progress can then result in atrophic gastritis, followed by intestinal metaplasia, which can finally result in dysplasia and adenocarcinoma (Correa, 1992) (Figure 4).

### Virulence Factors

**FIGURE 5:**
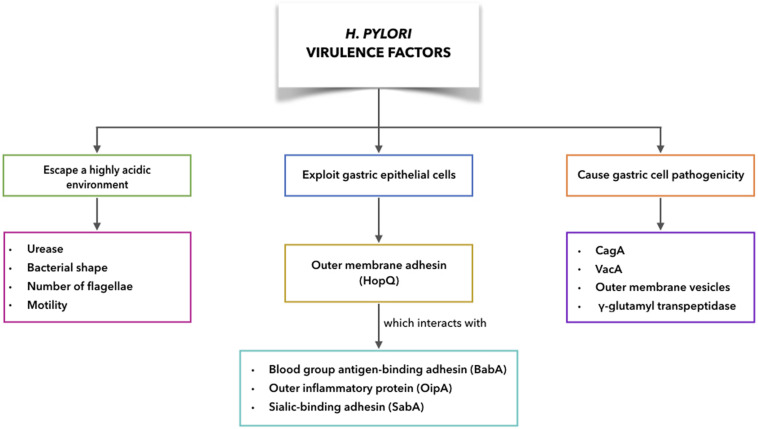
*H. pylori* virulence factors to escape acidic environment, to exploit gastric epithelial cells interacting with receptors on host cells, to cause gastric cell pathogenicity.

It has been shown that a number of the *H. pylori* virulence factors are particularly common for *H. pylori* strains associated with disease, compared to those that are not. *H. pylori* can escape from the harsh conditions of the gastric corpus and interact with gastric cells, while still evading host immune responses (Ansari and Yamaoka, 2017). This can result in more harm to the host due to the more direct exposure to *H. pylori* virulence factors. The need for eradication of *H. pylori* throughout the developing world is considered fundamental in the fight against non-cardia GC. In this respect, antibiotic therapy is the only weapon at our disposal at present, with bacterial antibiotic resistance now reaching alarming levels. Therefore, the development of a vaccine against *H. pylori* has become crucial. As knowledge of immune mechanisms active during *H. pylori* infection is required for successful vaccine development, as well as an awareness of the factors that allow *H. pylori* persistence, there follows an overview of both the *H. pylori* virulence factors (Figure 5) and the mechanisms of disease initiation.

### Virulence Factors to Escape a Highly Acidic Environment

Urease, bacterial shape, number of flagellae, and motility are the main contributing factors for *H. pylori* to escape from the harsh gastric conditions, which helps to establish persistent infections (Ansari and Yamaoka, 2019). After passing into the gastric lumen, *H. pylori* encounters a pH of ∼2.0. However, *H. pylori* has several factors that help it to interact with and survive within the gastric environment (Bui et al., 2016). *H. pylori* can produce large amounts of intracellular urease, and in addition, the bacterial cell surface will include extracellular urease following lysis in the stomach of some *H. pylori*. Thus urease-catalyzed hydrolysis of urea will result in ammonia and carbamate production, with the carbamate then spontaneously decomposing to produce further ammonia, and also carbonic acid. This carbonic acid can also be broken down to carbon dioxide and water (Marcus et al., 2005). With the ammonia in its protonated form, this can neutralize the acidity of the stomach, to provide a favorable microenvironment that remains nearly neutral around the *H. pylori* (Ansari and Yamaoka, 2019). At the same time, this ammonia production can disrupt the tight cell junctions in the stomach mucosa, where the loss of cellular integrity can lead to damage to the gastric epithelium. Carbon dioxide also protects *H. pylori* from bactericidal activities of metabolic products like nitric oxide (Ansari and Yamaoka, 2019). Moreover, a contribution of urease to tumor growth and metastatic dissemination through induction of angiogenesis has been shown recently (with the pre-existing vasculature showing formation of new blood vessels). Thus, it appears that urease has a key role in GC progression (Olivera-Severo et al., 2017).

On this basis, the development of a vaccine that uses urease as a target might represent the best choice in the fight against *H. pylori*. Liu et al. (2020) developed a vaccine candidate using a formulation of three of the commonly used *H. pylori* antigens, as neutrophil-activating protein, and the urease subunits A (UreA) and B (UreB). These were used in combination with a mucosal adjuvant and a double-mutant heat-labile toxin derived from *Escherichia coli*, which was evaluated in a mouse model of *H. pylori* infection for immunogenicity and therapeutic efficacy. These orally immunized *H. pylori*-infected mice showed significant reduction of gastric *H. pylori* colonization, as seen when determined at 2 and 8 weeks following immunization. This immunization also induced Th1/Th17 immune responses, indicating that this potential vaccine can overcome the mechanism of immune evasion of *H. pylori*, which restored the suppression of the Th2 immune response, and induced a strong humoral immune response (Liu et al., 2020).

Recently, Chen et al. (2020) focused on reduction of the required antigen levels, with immunization via three parenteral routes, combined with cyclic guanosine monophosphate–adenosine monophosphate (cGAMP) as adjuvant, to further promote the immunogenicity of this potential intranasal vaccine (Chen et al., 2020). However, regardless of the route of immunization, the previous attempts appear to be the first to define a way to eradicate *H. pylori* without using antibiotics. Indeed, they laid the foundation for development and optimization of an oral therapeutic *H. pylori* vaccine that can also provide long-term immunity. However, although these vaccines appear to be effective in mice, they remain to be tested in humans.

Considering again the *H. pylori* virulence factors, it has been shown that a mutation in bacterial cell shape determinants results in a straight rod morphology for *H. pylori*, which reduces the speed of *H. pylori* movement by 7–21%. This would suggest that the *H. pylori* helical shape is important for its penetration into the stomach mucosa and its movement within the viscous mucous layer (Ansari and Yamaoka, 2019). Despite the harsh conditions, the acid exposure also activates the production of proteins that help to further protect *H. pylori*. Indeed, the gastric niche activates flagellin and the flagellar proteins, and enhances *H. pylori* motility. These more motile cells might then reach a higher density on the gastric epithelium, which will trigger a greater inflammatory response (Ansari and Yamaoka, 2017).

### *H. pylori* Exploitation of Gastric Epithelial Cells to Increase Pathogenicity

The outer membrane proteins of *H. pylori* can interact with receptors on host epithelial cells, such as blood group antigen-binding adhesin (BabA), outer inflammatory protein (OipA), sialic-binding adhesin (SabA), and *H. pylori* outer membrane adhesin (HopQ). These interactions will have key roles in persistent *H. pylori* infection. The interactions with receptors on epithelial cells help to prevent *H. pylori* from being washed out during mucus shedding, while also providing nutrient access and promoting delivery of *H. pylori* bacterial toxins and further effector molecules into the cells of the host (Ansari and Yamaoka, 2017).

BabA has a particularly crucial role in *H. pylori* attachment, and provides significant contributions to *H. pylori* host-cell pathogenicity (Ansari and Yamaoka, 2017). Indeed, in countries in the West, infections arising from BabA-producing *H. pylori* strains are known to be associated with higher risk of development of peptic ulcer disease (Gerhard et al., 1999).

Moreover, several studies have reported associations between the expression of another outer membrane protein, SabA, and greater risk for chronic gastritis (Yamaoka et al., 2006), corpus atrophy, intestinal metaplasia, and GC (Ansari and Yamaoka, 2019). In addition, a further virulence protein of the *H. pylori* outer membrane protein family is encoded by the *OipA* gene. The expression of OipA is seen in particular for *H. pylori* strains that are adhered to gastric epithelial cells that show mucosal damage, and that are also associated with apoptosis of the host cells, and with IL-8–induced duodenal ulcers, and GC development (Yamaoka et al., 2002). A recent study highlighted the association of the *OipA* gene with development of chronic gastritis, whereby in countries in the western world, *H. pylori* strains that express OipA are often isolated from patients with GC (Sallas et al., 2019).

In addition, analysis of HopQ sequences from different *H. pylori* strains has defined the presence of two genotypes: type I and type II. In *H. pylori*, HopQ contributes to adherence of the bacteria to stomach epithelial cells through interactions with members of the family of carcinoembryonic antigen-related cell adhesion molecules (CEACAMs). Expression of these CEACAMs on the gastric epithelium cells is specifically associated with development of gastritis and GC (Ansari and Yamaoka, 2019). Recently it was shown that the HopQ and CEACAM interactions can promote gastric colonization of *H. pylori* and translocation of CagA into the gastric epithelium, which thus induces GC (Bonsor et al., 2018). Moreover, these HopQ and CEACAM interactions have an important role in inhibition of immune cell activity (Gur et al., 2019).

### Virulence Factors Associated With Gastric Cell Pathogenicity

#### Cytotoxin-Associated Gene A: CagA

*Helicobacter pylori* contains a pathogenicity island, cagPAI, as an extended chromosomal region where 32 genes are located that encode the multicomponent bacterial type IV secretion system (T4SS), which includes the effector protein CagA (Tegtmeyer et al., 2011). The CagA protein is 125–145 kDa, and is expressed by cagPAI-positive, and highly virulent, *H. pylori* strains; however, CagA is not expressed by cagPAI-negative, and less virulent, *H. pylori* strains (Ansari and Yamaoka, 2019). A syringe-like structure is formed by T4SS, which is required for transfer of the bacterial components into the host epithelial cells, which include CagA and peptidoglycan. Considering the abundant amounts of CagA that are synthesized by *H. pylori*, relatively little is translocated into host epithelial cells. The regulation of this translocation also involves autophagy and the ubiquitin-proteasome system, for its degradation (Ansari and Yamaoka, 2019). However, most people colonized with CagA^+^ *H. pylori* strains do not develop gastric cancer, thus implying that other microbial effectors may play an important role in carcinogenesis process (Varga et al., 2016). In this regard, high expression of polymorphisms in the gene of toll-like receptor 9 (TLR9), an innate immune receptor which detects DNA motifs located in microbial genomes, has been associated with the onset of premalignant lesions in the stomach. The molecular pathogenesis underlying this evidence is based on the ability of *H. pylori* cancer-associated *cag*T4SS to activate TLR9 through a contact-dependent mechanism between pathogen and host, and to transfer microbial DNA (Varga et al., 2016).

Moreover, inflammation of the gastric mucosa caused by *H. pylori* infection and the subsequent humoral and cell immune responses are associated with development of peptic ulcers and development of gastric cancer (GC) and mucosa-associated lymphoid tissue (MALT) lymphoma. *H. pylori* strains positive for cagPAI have been implicated in release of increased levels of inflammatory cytokines from host cells. These include IL-8 in particular, which is an important factor in peptic ulcer and GC immunopathogenesis (Crabtree and Lindley, 1994).

CagT is an essential component of cagPAI, and is important in the translocation of CagA into epithelial cells. CagL is another T4SS component with expression on the surface of the *H. pylori* cells. As well as having an important role in CagA translocation, CagL appears to be involved in *H. pylori* induction of IL-8 expression through the transforming growth factor-α/activated epidermal growth factor receptor (EGFR) signaling pathway (Kao et al., 2016).

#### Vacuolating Cytotoxin: VacA

Vacuolating cytotoxin (VacA) is an important cytotoxin that has a crucial role in *H. pylori* pathogenicity (Ricci, 2016). Initially, the 140 kDa pro-toxin of VacA is synthesized, with the 88 kDa mature form of the secreted toxin then undergoing proteolysis into two fragments: p33 and p55 (Atherton et al., 1995). Significant sequence variation is seen for the three heterogenic regions of VacA. Combinations of different sequences in these three regions can result in vacuolation. Moreover, there are three different genotypes: one that shows high vacuolating activity, another with intermediate activity, and the third with no vacuolating activity (Ansari and Yamaoka, 2019). Li et al. (2016) reported on a meta-analysis that showed that a VacA antibody is associated with peptic ulcer disease and risk of GC, and proposed that VacA be considered as a biomarker here (Li et al., 2016).

Moreover, a review reported recently that bacterial survival promoted by VacA is independent of accumulation of CagA. The transient receptor potential membrane channel mucolopin (TRPML) 1 activity that is associated with *H. pylori* VacA can inhibit the lysosomal and autophagic killing of bacterial cells, to allow establishment of an intracellular niche by the bacterial, where they can survive (Capurro et al., 2019).

#### Outer Membrane Vesicles

Outer membrane vesicles (OMVs) start in the form of blebs with diameters of 20–300 nm. These are then released, as has been seen during the logarithmic growth of several Gram-negative bacteria, including *H. pylori* (Ansari and Yamaoka, 2019). The OMVs include various outer-membrane-specific phospholipids, such as phosphatidylglycerol, phosphatidylethanolamine, phosphatidylcholine, cardiolipin, and lipopolysaccharides, along with some bacterial virulence factors, such as proteases, adhesins, and toxins (Olofsson et al., 2010). Once these OMVs have been released, they undergo absorption into the gastric epithelial cells, whereby the pathogen is protected from the respiratory burst and reactive oxygen species (Ansari and Yamaoka, 2019). Gastric biopsy specimens have been shown to include intracellular and extracellular OMVs. They appear to have roles in promotion of infection, impairment of cellular function, and modulation of host immune defenses through the production of the immunosuppressive IL-10 (Ansari and Yamaoka, 2019). It would therefore appear relevant to determine the numbers and contents of OMVs to contribute toward the diagnosis and treatment of *H. pylori* infections. A new and promising method for the identification, quantification and characterization of OMVs is polychromatic flow cytometry (Puca et al., 2019).

A further characteristic of *H. pylori* is their formation of biofilms. These complex structures provide the cells with a protective matrix of extracellular polymeric substances, which include proteins, carbohydrates and DNA. The formation of biofilms thus promotes survival and persistence of bacteria (Percival and Suleman, 2014). At the same time, bacteria in biofilms are associated with increased recombination, which can increase bacterial virulence and their antimicrobial resistance (Grande et al., 2012).

The extracellular DNA that is associated with planktonic OMVs and biofilm OMVs shows differences that indicate that planktonic and biofilm growth of *H. pylori* have different roles during infection and disease. Indeed, this indicates that biofilm OMVs can provide a reservoir of bacterial genetic material. Therefore, OMVs might have roles in *H. pylori* infection, which indicates the need to understand their compositions and biological activities to better understand *H. pylori* pathogenesis (Grande et al., 2015). Indeed, in a physicochemical evaluation of OMVs, Grande et al. (2015) showed that bacteria can generate nanovesicles that contain DNA, which might be of clinical relevance. Such nanovesicles might also be exploited for development of pharmacological agents against biofilm formation by *H. pylori*.

#### *H. pylori*γ-Glutamyl Transpeptidase

*Helicobacter pylori* γ-GT catalyzes transpeptidation and hydrolysis to amino acids of the γ-glutamyl of glutathione and glutathione-conjugated compounds (Ansari and Yamaoka, 2019). *H. pylori* γGT generates reactive oxygen species, and higher γGT activity has been associated with *H. pylori* strains from patients with peptic ulcer disease and GC, which suggests that γGT has a role in the development of severe pathogenicity. A recent study revealed a role for γGT in increased VacA-dependent vacuolation in epithelial cells, which appears to be mediated through extracellular glutamine hydrolysis (Ling et al., 2015). Ammonia is then released, and this accentuates VacA-dependent vacuolation, thus resulting in more severe gastro-duodenal diseases from *H. pylori* with higher γGT activity (Ansari and Yamaoka, 2019).

## *Helicobacter Pylori*: Mechanism of Disease

### *H. pylori-*Mediated Autophagy and Precancerous Lesions

Autophagy is a way through which cells respond to stress stimuli, which include lack of nutrients and microbial infection (He and Klionsky, 2009). Through this conserved mechanism, eukaryotic cells are more able to maintain their cellular homeostasis.

Cellular components are stored in autophagosomes that are trafficked through the cell and can fuse with lysosomes for degradation and recycling. Infection of the AGS gastric adenocarcinoma cell line with *H. pylori* for 6 h resulted in autophagy that was dependent on VacA (Terebiznik et al., 2009). This suggested that autophagy is used by cells infected by *H. pylori* to avoid the damage induced by toxins, and thus to promote cell survival. In addition, Raju et al. (2012) reported that 24 h exposure to VacA disrupts the antiphagocytic pathway, with an accompanying cellular accumulation of defective autophagosomes. It has also been shown that *H. pylori* regulates autophagy and expression of autophagy-related genes in macrophages and gastric cells (Castaño-Rodríguez et al., 2017).

To summarize, during the early stages of cancer, autophagy acts as a control system, and if it is inhibited, this results in precancerous lesions and the production of reactive oxygen species. These can, in turn, promote the development of cancer characteristics, which include promotion of cell growth, invasion, and metastasis formation. In GC, this would contribute to progression of precursor lesions (Valenzuela et al., 2015).

### *H. pylori-*Associated Inflammation

Chronic inflammation is involved in the pathogenesis of several cancers, and it is particularly important for *H. pylori*-associated GC (Valenzuela et al., 2015). Indeed, maintained gastric mucosa inflammation will result in the production of high levels of nitric oxide. This will contribute to the damage of nucleotide bases and to transcriptional regulation, as it increases DNA methyl transferase activity (Huang et al., 2012). Indeed, for the *E-cadherin* tumor suppressor gene, its promoter region has often been seen to be hypermethylated in *H. pylori* infections of adult patients, and its transcriptional activity will be altered in gastric cells (Perri et al., 2007).

Inflammatory responses have been shown to be controlled by bacterial virulence factors. *H. pylori* virulence factors, such as the VacA and CagA, can inhibit T-cell activation and evade recognition by Toll-like receptors (Mejías-Luque and Gerhard, 2017). On the other hand, γGT can promote oxidative stress and enhance apoptosis of gastric cells (Valenzuela et al., 2015). These responses are balanced by the intrinsic host-specific mechanisms. This is supported in knock-out animal models, where the importance of host factors for the modulation of inflammation has been shown, such as the anti-inflammatory effects associated with TLR9 signaling (Valenzuela et al., 2015).

### *H. pylori* and Oxidative/Nitrosative Stress

The oxidative stress generated in the gastric mucosa during *H. pylori* infection is crucial to gastric carcinogenesis. The consequences of reactive oxygen species are evident in changes in lipid and protein expression and in damaged DNA (Valenzuela et al., 2015). Moreover, anti-oxidant capacity decreases because of lower levels of antioxidant molecules, such as glutathione (GSH) in the gastric mucosa of *H. pylori*-infected patients (Valenzuela et al., 2015). Furthermore, in the infected mucosa, nitric oxide is generated in the lymphocytes, macrophages, and gastric cells by inducible nitric oxide synthetase. This results in the production of the highly toxic peroxynitrite, and the consequent damage to proteins and DNA through the generation of nitrotyrosine and DNA adducts, respectively (Sakaguchi et al., 1999; Cherdantseva et al., 2014; Valenzuela et al., 2015). However, this oxidative stress induced by *H. pylori* infection of gastric cells can be attenuated through the production of scavenger molecules, such as metallothionines, a role for which has been demonstrated in animal models (Mita et al., 2008).

### *H. pylori* and ErbB Receptor Family

The ErbB family of proteins comprises four receptor tyrosine kinases: EGFR (ErbB1/HER1), ErbB2 (HER2/neu), ErbB3 (HER3), and ErbB4 (HER4). When these receptors are activated, they can form homo-dimers or hetero-dimers, which is followed by activation of phosphorylation cascades within the cell. This, in turn, leads to activation of the “Akt-mammalian target of rapamycin” (mTOR) PI3K and Ras–Raf mitogen-activated protein kinase (MAPK)/ extracellular signal-related kinase (ERK) pathways. These represent important mechanisms for proliferation and survival of cancer cells (Yarden and Pines, 2012).

The EGFR has been reported to be overexpressed in up to 64% of gastric tumors, with its oncogenic role in GC well known. It has also been suggested that high gene amplification of the EGFR is related to poor patient outcome. However, a 2013 meta-analysis that compared five studies that included 1,600 patients reported that in GC, EGFR expression was not an independent predictor of survival (Arienti et al., 2019).

ErbB2 amplification/overexpression has been reported for 6–30% of GCs, with this variability attributable in part to histological subtypes and primary tumor location (Boku, 2014). In a comparison with diffuse-type tumors, ErbB2 overexpression showed greater prevalence for intestinal-type and gastroesophageal junction tumors (Zheng et al., 2010; Grávalos et al., 2011). Many studies have shown ErbB2 positivity to be indicative of poor patient prognosis; however, other studies have also not observed any association between ErbB2 status and patient outcome (Janjigian et al., 2012). Nevertheless, ErbB2 expression and gene amplification are used as a biomarker for targeted therapy of patients with GC (Arienti et al., 2019). The monoclonal antibody trastuzumab binds to the ErbB2 extracellular domain, through which it blocks ErbB2 receptor cleavage, inhibits its dimerization, and induces antibody-dependent cellular cytotoxicity. In the phase III ToGA trial, the combination of trastuzumab with standard cisplatin and 5-fluorouracil therapies improved overall survival from 11.1 to 13.8 months for patients with ErbB2-amplified gastric adenocarcinomas (Bang et al., 2010).

ErbB3 and ErbB4 can be mutated in GC, although at low frequency (<10%; Jaiswal et al., 2013; Chen et al., 2015). Nielsen et al. (2014) demonstrated down-regulation of both ErbB4 and NRG4, its specific ligand, in cancer tissues. This was accompanied by increased EGFR signaling through up-regulation of its ligands, and in particular, up-regulation of ErbB2.

Ectopic CagA expression in human gastric cell lines (e.g., MKN1, AGS, HFE-145 cells) induces significant increases in ErbB2 expression and gene copy numbers (Shim et al., 2014). This study concluded that CagA appears to be a candidate biomarker for ErbB2 amplification, and might promote development of GC through induction of ErbB2 amplification and overexpression (Shim et al., 2014). In contrast, the CagA effects on cell scattering and elongation are antagonized by VacA through inactivation of the EGFRs, including ErbB2, and their downstream signaling, which leads to suppression of MAPK activity and consequently of the “hummingbird” phenotype. These results suggest a novel mechanism whereby *H. pylori* can modulate, and thus avoid, the damage to the cells of the gastric epithelium (Tegtmeyer et al., 2009).

## Treatment

Patients with *H. pylori* infection can at present be treated with a number of antibiotic regimens. The recommendations in the 2007 American College of Gastroenterology guidelines indicate first-line treatment with 10–14 days of the standard triple therapy of a proton pump inhibitor (PPI) with amoxicillin and clarithromycin. However, recent years have seen the development of clarithromycin resistance worldwide, with decreasing *H. pylori* eradication rates. Although there are limited data on clarithromycin resistance in the United States, this has been estimated as up to 30%. Consequently, alternative regimens are urgently needed to reverse this increase in resistance to clarithromycin (Kamboj et al., 2017).

One regimen involves sequential therapy initially with 5 days of a PPI and amoxicillin, followed by a further 5 days of a PPI with metronidazole and clarithromycin. This sequential therapy is based on the concept that amoxicillin can first weaken the bacterial cell wall, to then allow metronidazole and clarithromycin to act directly on the bacteria, while the efflux of the antibiotics through the bacterial drug-efflux channels is prevented. Although this appears to make sense, the reverse of this sequential therapy (i.e., PPI, clarithromycin, and metronidazole before PPI and amoxicillin) was shown to have a similar eradication rate (Kamboj et al., 2017).

As well as the use of the quadruple concomitant PPI, bismuth, metronidazole, and tetracycline (PBMT) therapy, another regimen proposed involves a non-bismuth combination therapy, as a PPI with amoxicillin, metronidazole, and clarithromycin (PAMC). The idea here is simply that with the addition of the fourth antibiotic (compared to standard triple therapy) higher eradication rates should be obtained. Furthermore, a systematic review from the Cochrane database investigated the optimal treatment duration for *H. pylori* infection, and this indicated 14 days of therapy as ideal. In their comparison with 7 days regimens, the extension to 14 days of treatment provided significantly greater eradication rates (72.9 vs. 81.9%, respectively), and showed the relative risk of *H. pylori* persistence as 0.66 (95% confidence interval, 0.60–0.74) (Kamboj et al., 2017).

On the basis of increased clarithromycin resistance worldwide, and bearing in mind that there is information available for local antibiotic resistance patterns, new guidelines have been proposed. The 2016 Toronto Consensus guidelines thus recommended first-line treatment as 14 days of either the appropriate PAMC therapy or the bismuth PBMT quadruple therapy (Kamboj et al., 2017).

The treatment of *H. pylori* infection now also includes the new synthesized silver ultra-nanoclusters (SUNCs) alone or in combination with metronidazole (Grande et al., 2020). The development of biofilms by bacterial populations represents the main virulence factor in many localized chronic infections (Koo et al., 2017). The hypothesis behind the SUNC effectiveness for eradication of *H. pylori* infection relies on the ability of the nanoparticles to penetrate through the biofilm extracellular polymeric substances matrix: SUNCs can then disrupt the biofilm to induce cell death, and also promote cell detachment from the surface, and reduce drug resistance (Grande et al., 2020). These SUNCs also provide the possibility of carriers for drug delivery (Grande et al., 2020).

## Pros and Cons of *Helicobacter Pylori* Treatment

As part of the analysis of the options for treatment of *H. pylori* infection, it should also be asked whether there is the necessity for eradication therapy, given that its role remains debatable to date. In 1989, Strachan proposed the “hygiene hypothesis” (Strachan, 1989), a theory that suggested that the more recent improved hygiene in early life reduces microbial exposure. This reduced exposure removes the early priming of the immune response, and thus the protection against atopic disorders, asthma, and autoimmune diseases. More specifically, this immunological theory states that the T-helper 1 (Th1) and Th2 cells need to be in balance for correct functioning of the immune system. If Th1 cells decrease because of a lack of exposure to microorganisms during development, there will be an increase in Th2 cells, which will result in more Th2-related reactions, such as allergies (Bae, 2018).

Several mechanisms have been suggested that can provide a link between the hygiene hypothesis and *H. pylori* infection (Cremonini and Gasbarrini, 2003). Indeed, *H. pylori* influences naïve T-cell induction through the two main functional subsets, as the Th1 and Th2 cells. In *H. pylori*-infected patients, T cells of the gastric mucosa produce more interferon-γ and less IL-4 compared to those of uninfected patients. This suggests that *H. pylori* infection can lead to a Th1-polarized immune response, as IL-4 is a cytokine that induces characteristic Th2 responses (Bamford et al., 1998; Sommer et al., 1998). On this basis, through suppression of the Th2 response, *H. pylori* infection might also decrease the risk of asthma and allergies (Ness-Jensen et al., 2019). Therefore, those in support of the hygiene hypothesis believe that it will not only be the relatively bacteria-free environment in early life, but also the use of antibiotics that increases the risk of autoimmune disorders in later life (Miftahussurur et al., 2017). At the same time, *H. pylori* has been shown to protect against esophageal adenocarcinoma (Xie et al., 2013).

So, is it really necessary to eradicate *H. pylori*? This is a controversial issue that needs better examination considering that there are some diseases in which *H. pylori* appears to have an etiopathogenetic role, although this has not been completely demonstrated.

Despite disagreement, *H. pylori* eradication in the western world is still crucial in the fight against antral GC and to prevent peptic ulcers, gastritis, and mucosa-associated lymphoid tissue lymphoma. First, *H. pylori* has to be detected serologically, for example using enzyme-linked immunosorbent assays or the urea breath test, or endoscopically (followed by biopsy). This last process is a more invasive option, but it also has better sensitivity. Moreover, there still remains the need to define the use of non-invasive methods in screening for GC, such as *H. pylori* serology, serum trefoil factor 3, and pepsinogen tests, as their use has been controversial (Rawla and Barsouk, 2019). Secondly, even if treatment is generally considered necessary for those with chronic infections, antibiotic therapy remains controversial for asymptomatic patients, who constitute the majority.

After a more detailed evaluation of the *Pros* and *Cons* for *H. pylori* eradication (Table 1), knowledge of the microbiota will be proposed as a new readout key on the basis of its meaningful role in therapeutic strategies.

**TABLE 1:** Relevant literature on the *Pros* and *Cons* of *H. pylori* infection.

Argument	Conditions	References	
		Title	Citation
*Pros*	Gastric cancer prevention	*Helicobacter pylori*: the past, present, and future in management	Kamboj et al., 2017
	Peptic ulcer prevention	*Helicobacter pylori*: the past, present, and future in management	Kamboj et al., 2017
	Better absorption of drugs in thyroid diseases	*Helicobacter pylori* infection and drugs malabsorption	Lahner et al., 2014
*Cons*	Asthma and allergy	*Helicobacter pylori* in relation to asthma and allergy modified by abdominal obesity: the HUNT study in Norway	Ness-Jensen et al., 2019
	Esophageal cancer	*Helicobacter pylori* infection and esophageal cancer risk: an updated meta-analysis	Xie et al., 2013
	Barrett’s esophagus	*Helicobacter pylori* infection and short-segment/long-segment Barrett’s esophagus in a Japanese population: a large cross-sectional study	Usui et al., 2020

### Pros of Treatment Against *H. pylori*

#### Prevention of Peptic Ulcers and Gastric Cancer

As we have already discussed, *H. pylori* is the most common cause of duodenal and gastric ulcers. Infection with *H. pylori* results in a 10–20% lifetime risk of peptic ulcers, with a 1–2% risk of GC. Infection with *H. pylori* strains that express CagA results in higher risk of peptic ulcers and GC, because of the associated increased inflammation. Thus, it can be inferred that it is crucial to recognize *H. pylori* infection and its relation to clinical circumstances, as morbidity can be greatly decreased through the relevant treatment (Kamboj et al., 2017).

#### Better Absorption of Drugs in Thyroid Diseases

For the efficacy of any oral drug treatment, an important factor is the drug absorption (Lahner et al., 2014). There are a number of key processes that can determine the bioavailability of a drug in any particular patient: the gastric pH, the time for gastric emptying, the mucus layer and mucosa condition and the mucosa permeability, the drug transit time, and the composition of the local microflora (Lahner et al., 2020). Lahner et al. (2014) demonstrated a causal relationship between *H. pylori* infection and reduced absorption of orally administered drugs. Furthermore, poor bioavailability of L-thyroxine has been reported for *H. pylori*-related disorders. Although the mechanisms have not yet been clarified, L-thyroxine (T4) absorption appears to be affected by the pathological changes to the intragastric environment that are associated with *H. pylori* infection (Lahner et al., 2014). For thyroid failure, and also for differentiated thyroid carcinoma, the drug of choice is T4. This treatment requires the optimum required daily dose to be calculated, which is based on the patient’s anthropometric characteristics, and assumes their efficient absorption of T4 in the intestine. However, both gastric and intestinal disorders can have adverse effects on T4 absorption (Virili et al., 2019), although this takes place in the small intestine (Lahner et al., 2014). This can occur through different mechanisms. Achlorhydria is a feature of gastric atrophy that can result in long-lasting *H. pylori* infection, and it reduces the dissolution rate of T4 tablets. Then villous atrophy can significantly reduce both the absorptive surface to T4 and the number of transporters of T4 (Virili et al., 2019). The gut microbiota also appear to bind iodothyronines, and can deconjugate their sulfo-conjugated and/or glucurono-conjugated products (Virili et al., 2018), which converts these in their naïve forms that can be reabsorbed through the enterohepatic circulation.

Centanni et al. (2006) demonstrated that gastric atrophy causes the need for increased T4 dosing, with the similar effect on T4 treatment of infection with *H. pylori* can be reversed by eradication of these bacteria. These data were supported by Checchi et al. (2008), who demonstrated positive correlation between the increased dosing of T4 and higher serum levels of parietal cell antibodies. These antibodies target H^+^/K^+^ ATPase, which shares some epitopes with *H. pylori*, and they are considered to be a marker of gastric autoimmunity (Lahner et al., 2008). However, the increased T4 dosing required is greater for histologically proven gastric atrophy than it is for parietal-cell-antibody-positive patients, which indicates that the presence of these parietal cell antibodies is not predictive for atrophic gastritis (Lahner et al., 2014). Of note, for patients with thyroid autoimmunity, the co-presence of T4 malabsorption and chronic unexplained anemia should prompt diagnostic workup for gastric disorders, in that this sign is shared by both *H. pylori* infection and gastric atrophy (Sibilla et al., 2008).

### Cons of Treatment Against *H. pylori*

#### *H. pylori*, Asthma and Allergy: An Inverse Relation

The second Nord-Trøndelag Health Study (HUNT2; Norway) was a population-based study of 10,005 individuals that was performed from 1995 to 1997. *H. pylori* infection was defined using an enzyme immunoassay (Pylori EIA-IgG tests), with the recording also of height, weight, and waist circumference. Questionnaires provided information on self-reported asthma and allergic diseases. Overall, infection with *H. pylori* was not associated with asthma or allergic diseases. However, according to the stratification of waist circumference to define abdominal obesity, these patients showed 30–40% reduced risk of asthma, and 25% reduced risk of allergic diseases when infected with *H. pylori* (Ness-Jensen et al., 2019). From this, it can be concluded that in individuals with abdominal obesity, infection with *H. pylori* results in reduced risk of asthma and allergic diseases. This would suggest a causal pathway from reduced *H. pylori* infections due to obesity, to an increased risk of asthma and allergic diseases (Ness-Jensen et al., 2019).

#### *H. pylori* Infection and Esophageal Cancer Risk in Populations in the East

A meta-analysis in 2013 indicated that infection with *H. pylori* can significantly decrease the risk of esophageal adenocarcinoma in populations in the West (Xie et al., 2013). In terms of the risk of esophageal squamous cell carcinoma (ESCC) instead, there was no significant association for the pooled populations from the East and West. Further, when stratified for study location, there was again no significant association between *H. pylori* infection and ESCC risk for populations from the West. However, *H. pylori* infection showed significant association with decreased risk of ESCC in populations in East Asia (Xie et al., 2013).

This phenomenon can potentially be explained in a number of ways. First, the carcinogenesis pathways between ESCC and esophageal adenocarcinoma show fundamental differences. The known risk factors for ESCC are alcohol consumption, cigarette smoking, hot-temperature food, salty food, nutrient deficiency, low vegetables intake, pickled vegetables, chronic mucosal irritation, and history of cancer; esophageal adenocarcinoma is closely related to Barrett’s esophagus (Xie et al., 2013). Secondly, different effects might arise from genetic differences across ethnic groups. Indeed, in a meta-analysis by Umar et al. (2013), they showed that a polymorphism in phospholipase C-ε1 resulted in significant risk for gastric and esophageal tumors in Asian (Chinese) populations, although this was not seen for Caucasians. In the populations from the East, the esophageal adenocarcinoma incidence rates were higher in urban areas, which corresponded to where the diet and lifestyle are more closely related to those in countries in the West. Thus, when nutritional intake and lifestyle are combined with infection with *H. pylori*, this has parallel effects for populations from the East and West. In contrast, patients with ESCC were mainly those in developing countries in the East, where lack of nutrients and the intake of hot beverages are more common than for populations in the West. These different aspects might thus influence any protective effects against infection with *H. pylori* (Umar et al., 2013).

#### *H. pylori* Infection and Short-Segment/Long-Segment Barrett’s Esophagus

Usui et al. (2020) carried out a cross-sectional study in a Japanese population to investigate any relationships between *H. pylori* infection and short-segment and long-segment Barrett’s esophagus. The study subjects included 36,615 asymptomatic Japanese individuals. Seropositivity for *H. pylori* was associated with a significantly lower rate of long-segment Barrett’s esophagus (odds ratio, 0.42) and a significantly higher rate of short-segment Barrett’s esophagus (odds ratio, 1.66). Thus, in this Japanese population, there was an inverse association between *H. pylori* infection and long-segment Barrett’s esophagus, while *H. pylori* infection was significantly associated with short-segment Barrett’s esophagus only in those individuals without any reflux esophagitis. Therefore, infection with *H. pylori* appears to be a risk factor for short-segment Barrett’s esophagus, especially for individuals without reflux esophagitis (Usui et al., 2020).

### Beyond the Hygiene Hypothesis: Are the Microbiota the Key?

Originally, the hygiene hypothesis indicated that when infections are caught in early life through unhygienic contact, such as fecal-oral viral infections, this can prevent allergies and asthma. Later on, this theory was modified; rather than limiting it to infections, the western lifestyle alters the colonization of the infant gut by microorganisms beyond pathogens, such as commensals and symbionts, which have their roles in the development of a large number of diseases (Ege, 2017).

Nowadays, the body microbiota are known to be fundamental in the induction and function of the immune system, and multiple means have evolved to maintain the symbiotic relationship between the immune system and the microbiota. The preservation of this “dialogue” allows the development of protective responses to pathogens and the use of regulatory pathways involved in tolerance to innocuous antigens. In particular, the gut microbiota consist of tens of trillions of microorganisms and weighs up to 2 kg; these microorganisms are now believed to influence health as well as disease. Until recently, it was believed that the microbiota are located only in the small bowel and colon, as the stomach was widely considered as sterile. Then, the identification of *H. pylori* focused the attention on the gastric microbiota, as “an ecological niche for bacteria” (Sgambato et al., 2017).

Recent data have shown that there is a large diverse bacterial community in the stomach, where the colonization densities are believed to be from 10^1^ to 10^3^ colony forming units/g (Wroblewski and Peek, 2016). The gastrointestinal microbiota contribute to different biological functions, which have been shown to include: protection against colonization by pathogens; digestion of complex carbohydrates; maturation of the immune system; and regulation of the central nervous system. More evidence has also become available that indicates roles for the gut microbiota in diseases, including for obesity and diabetes, and also for inflammatory bowel disease (Alarcón et al., 2017). The precise mechanisms that determine the compositions of the gastric bacteria of any individual are not well understood, although the main effectors in the modulation of the gastric niche appear to be diet, use of antibiotics, probiotics, PPIs, and H2-antagonists, and *H. pylori* infection. The host immune status can also regulate the gastric ecology, as a good immune response contributes to the control of dysbiosis (Espinoza et al., 2017).

What interests us most here is that within this framework, the complex gastric microbiota influence homeostasis and disease, especially in combination with *H. pylori* (Wroblewski and Peek, 2016). Bik et al. (2006) showed that individuals who are *H. pylori* negative can have widely diverse gastric microbiomes. In their sequencing of 1,833 bacterial clones taken from 23 gastric biopsies they identified 128 phylotypes that belonged to eight bacterial phyla. The top five of these for abundance were *Proteobacteria*, *Firmicutes*, *Bacteroidetes*, *Fusobacteria*, and *Actinobacteria*. However, for individuals positive for *H. pylori* infection, it was *H. pylori* that was the most abundant phylotype (Bik et al., 2006). Indeed, in a further study of *H. pylori*-colonized patients, *H. pylori* accounted for 93–97% of the sequence reads, with only 33 phylotypes detected; conversely, 229 more phylotypes were detected in *H. pylori*-negative individuals (Andersson et al., 2008).

To summarize, the data available to date show that *H. pylori* colonization leads to greatly reduced diversity of the gastric microbiota. Moreover, Yu et al. (2014) showed that low microbial diversity of the upper digestive tract was associated with a low serum pepsinogen I/pepsinogen II ratio, which has also been implicated in gastric carcinogenesis. Consistent with these studies, Liu et al. (2019) also analyzed the gastric mucosal microbiota of different microhabitats in the stomach of 276 patients who were enrolled without preparatory chemotherapy. Here, there was a decreasing trend of bacterial richness going from the normal tissue to peritumoral and tumoral tissues, which indicated that as the microenvironment of a tumor is altered, it becomes unsuitable for colonization with specific bacterial. Moreover, bacteria from the different stomach microhabitats showed different correlation networks and functions (Liu et al., 2019). So, although a role for *H. pylori* in gastric carcinogenesis cannot be questioned, accumulating evidence indicates that other bacteria in the gastric microbiota also appear to be responsible for transformation of stomach epithelial cells (Liou et al., 2019). However, it remains to be clarified whether it is the *H. pylori* infection that promotes growth of unwanted microorganisms, or *vice versa*.

Espinoza et al. (2017) showed that an altered microbiota can create favorable conditions for *H. pylori* colonization, which in turn, supports the growth of certain bacteria, such that the consequent gastric dysbiosis might promote GC. In this regard, in their investigation with transgenic insulin-gastrin mice, Aviles-Jimenez et al. (2014) reported that while mono-infection with *H. pylori* promoted gastritis and the development of GC, for the *H. pylori*-infected mice with an abundant gastric microbiota, less severe gastric lesions were seen, along with delayed onset of GC. This thus indicated that among the microbes in the gastric microbiota, there were some that might contribute to carcinogenesis, potentially through promotion of the production of nitrite and accumulation of carcinogenic N-nitroso compounds (Aviles-Jimenez et al., 2014; Sgambato et al., 2017). In a survey carried out more recently, Jo et al. (2016) stated that in patients with GC, the numbers of nitrosating/nitrate-reducing bacteria besides *H. pylori* were at least twice that of healthy individuals with the same *H. pylori* status, although this did not reach statistical significance. Therefore, to be able to state with more certainty and scientific significance whether changes in the gastric microbiota are crucial to the development of GC or are secondary to the resulting changes in the gastric environment, further detailed molecular studies need to be carried out.

It is important to note that microbial infections have for a long time now been considered to be responsible for a range of tumor types, through modulation of host-cell transformation, and hence by promotion of malignant features and inflammation, and disruption of cell integrity through genomic or epigenetic modifications. So, the concept that the gut microbiota have a role in the development of GC does not appear to be particularly strange (Eyvazi et al., 2020). Moreover, under the assumption that *H. pylori* has a relevant role in Correa’s cascade (i.e., from non-atrophic gastritis to atrophic gastritis, and further to intestinal metaplasia, dysplasia and adenocarcinoma), it can be stated that the inflammatory process of gastritis is initiated and sustained by *H. pylori* infection, which can exist for decades preceding any malignant transformation. Eventually, this transformation might arise due to increased gastric pH, since parietal cells are lost and there can then be overgrowth of different, non-*Helicobacter* microbiota (Alarcón et al., 2017). Indeed, many differences have been shown across the microbiota of patients with GC, intestinal metaplasia, and chronic gastritis. This suggests that the commensal flora of the gut have an important role in the carcinogenesis of *H. pylori*. In contrast, gradual changes in gastric acidity might also be seen to arise from the histological changes that can be induced by *H. pylori*, such that other microorganisms might be facilitated in terms of their colonization of the gastric epithelium (Alarcón et al., 2017).

Ferreira et al. (2018) demonstrated that the main genera enriched in the microbiota of patients with GC were *Achromobacter*, *Citrobacter*, *Lactobacillus*, *Clostridium*, *Rhodococcus*, and *Phyllobacterium*. However, future studies will need to be designed to identify which of the bacterial taxa in the stomach are actually predictive of gastric diseases, considering also the specific disease stages defined by Correa’s cascade. Thus, it is hoped that it will become possible to manipulate the specific microbiota of an individual to provide more favorable outcomes following infection with *H. pylori*. Under this premise, it can be suggested that beneficial microbes might indeed restore the gastric microbiota, and thus provide an effective therapeutic.

As reported by Poore et al. (2020), characterization of the cancer microbiome will allow the development of techniques that can be used to exploit non-human, microorganism-derived molecules for diagnosis of the major human diseases. Genome and transcriptome sequences of 33 types of cancers of treatment-naïve patients taken from The Cancer Genome Atlas were examined for microbial reads. They reported that there were unique microbial signatures for tissue and blood samples within and between most of the major cancer types, whereby the blood signatures remained predictive for patients with cancers of stage Ia–IIc. Using solely the plasma-derived data, they discriminated between the cell-free microbial nucleic acids from the samples from healthy and cancer-free individuals (*n* = 69) and the samples from patients with multiple cancer types (*n* = 100; mainly prostate, lung, melanoma) (Poore et al., 2020). It appears clear that this method will become a particularly useful microbiome-based oncological diagnostic tool.

Nowadays, the move from studies that are descriptive to functionally based studies that are designed to investigate the effects on the gastric mucosa of specific taxa and bacteria-derived metabolites will provide improved understanding of the mechanisms behind dysbiosis-associated genotoxicity and inflammation. That is why attention is being focused on the role of probiotics, as these might prove to be effective for rebalancing the composition of the human microbiota. Probiotics have been defined as “live microorganisms which, when administered in adequate amounts, confer a health benefit on the host” (Homan and Orel, 2015; Schulz et al., 2015), and they are used for the treatment of *H. pylori* infections, although generally as an addition to antibiotics treatments. Probiotics are used in this way for two different reasons: to increase the rate of *H. pylori* eradication, and to reduce adverse gastrointestinal events that can arise from antibiotic treatments. Although probiotics can be used as a monotherapy, this remains on an experimental basis, and at present they are used together with standard eradication therapies (Alarcón et al., 2017).

The influence of the combination of probiotics with antibiotics on the gut microbiota composition was investigated by Oh et al. (2016). They reported that for the gut microbiota of the individuals investigated, there were three phyla that showed the greatest abundance: *Firmicutes*, *Bacteroidetes*, and *Proteobacteria*. After their treatments with standard therapies vs. standard therapies plus the probiotics, *Firmicutes* decreased and *Proteobacteria* increased, although the level of this decrease and increase in the antibiotics alone group was greater than seen for the antibiotics plus probiotics group. Also, there were increased levels of antibiotic-resistant bacteria for the antibiotics only group, compared to antibiotics plus probiotics (Oh et al., 2016). These results are especially interesting because they show that therapy with antibiotics plus probiotics can avoid the development of antibiotic resistance, although further investigations are required here.

The protective effects of *H. pylori* gastric colonization against esophageal adenocarcinoma can also be explained in the light of studies on the gut microbiota. Hypochlorhydria induced by *H. pylori* appears to result from loss of parietal cell function, which is seen in particular for patients with more polymorphisms of IL-1β. However, the changes to the gastric microbiota that arise from absence of *H. pylori* might also cause an increase in the risk of development of esophageal adenocarcinoma (Abreu and Peek, 2014).

However, to determine with more certainty whether such perturbations in the gastric microbiota are crucial for development of GC or whether they are only secondary to the changes in the gastric environment, there remains the need for further detailed molecular studies, particularly in relation to the composition of the gastric microbiota.

## Concerns About Widespread Screening Methods

**FIGURE 6:**
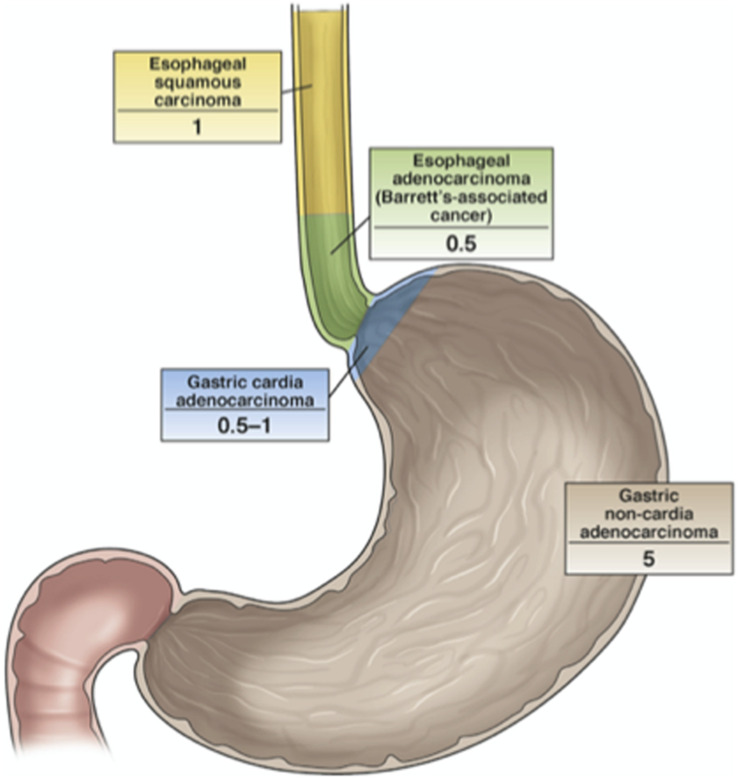
The relationship between *H. pylori* infection and cancers of the esophagus and stomach. The numbers indicate the odds ratios for the development of the defined cancers in *H. pylori*-infected vs. uninfected individuals. *H. pylori* is less common in patients with esophageal adenocarcinoma and gastric cardia adenocarcinoma, even though its protective role for esophageal cancer has not been demonstrated yet (Moss, 2016).

Even if *H. pylori* is globally considered one of the causes of antral GC, nowadays there is little effort to use widespread screening and eradication programs, for the reasons already explained here. In 2004, a pilot study that involved population screening was initiated on Matsu Island (Taiwan). The results were initially considered very promising, as cancer incidence decreased by approximately 25% (Moss, 2016). However, there was concern in terms of the potential ecological consequences of antibiotics use to prevent *H. pylori*-related diseases as applied to such a large proportion of a population. To avoid this, a non-antibiotic strategy would be preferred in the future. Indeed, a vaccination against *H. pylori* based on UreA and UreB and combined with neutrophil-activating protein and different adjuvants has suggested this possibility, although to date this has only been used for oral immunization of mice (Chen et al., 2020; Liu et al., 2020). At the same time though, it is important to consider the great reluctance for the adopting of this kind of approach, because it is believed that *H. pylori* can also have beneficial effects against esophageal disease. Indeed, a number of studies have shown *H. pylori* to be less common in patients with esophageal adenocarcinoma, as has already been discussed (Figure 6). However, such an inverse association for *H. pylori* and esophageal adenocarcinoma is not conclusive evidence that *H. pylori* will indeed be protective for the esophagus for all types of patients (Moss, 2016).

## Conclusion

Gastric adenocarcinomas, and especially antral ones, are linked to *H. pylori* infection, and the progression of *H. pylori* infection through chronic active gastritis to GC has been shown. Although most *H. pylori* infections do not show clinical symptoms, for the patients with long-term infections, 1–3% will suffer from gastric adenocarcinoma.

There is no real doubt that several mechanisms of tumorigenesis that are induced by *H. pylori* have important roles in the developing of GC, such as CagA, inflammation, and oxidative stress. Also, environmental and dietary factors are known to worsen *H. pylori*-induced carcinogenesis. Moreover, according to the theory of gut microbiota involvement in development of cancers, the presence of microbial metabolites and toxins, and the by-products of inflammation that can be produced by a dysbiotic microbiome, might induce host-cell damage directly, or might instead interfere with the signaling pathways of the host, such as to influence cell proliferation and survival (Pereira-Marques et al., 2019). So, even if *H. pylori* infection appears to invoke multiple patterns, and if GC results from a well-known multistep process, there remain many events in these tumorigenic processes that need to be clarified and further investigated. Indeed, although significant achievements in the prevention and treatment of GC have been made worldwide, GC still remains the fifth most commonly diagnosed cancer, and the third most deadly (Rawla and Barsouk, 2019).

In conclusion, given that *H. pylori* remains the most important risk factor for peptic ulcers and GC, its eradication might augment the risk of other diseases and of increased antibiotics resistance. Therefore, further investigations are required into the pathogenic patterns and the interactions of *H. pylori* with diet, genetics, and the gut microbiota.

## Author Contributions

All authors made direct and substantial contributions to the study, and have approved the manuscript for publication.

## Conflict of Interest

The authors declare that the research was conducted in the absence of any commercial or financial relationships that could be construed as a potential conflict of interest.
